# Clinical and Genetic Analysis in Neurological Wilson’s Disease Patients With Neurological Worsening Following Chelator Therapy

**DOI:** 10.3389/fgene.2022.875694

**Published:** 2022-04-04

**Authors:** Haiman Hou, Dingbang Chen, Junxiu Liu, Li Feng, Jiwei Zhang, Xiuling Liang, Yuming Xu, Xunhua Li

**Affiliations:** ^1^ Department of Neurology, The First Affiliated Hospital of Zhengzhou University, Zhengzhou, China; ^2^ Department of Neurology, The First Affiliated Hospital of Sun Yat-Sen University, Guangzhou, China; ^3^ The First People’s Hospital of Zhongshan City, Zhongshan, China

**Keywords:** ATP7B, mutational analysis, chelators, neurological worsening, Wilson’s Disease

## Abstract

**Objectives:** None of the previous studies have focused on the genetic effect on neurological worsening in neurological Wilson’s disease (WD) patients following chelator therapy. We aimed to evaluate the clinical and genetic role in the occurrence of neurological worsening.

**Methods:** We retrospectively reviewed the medical records of neurological WD patients who received initial chelator therapy and genetic test. Clinical, laboratory, and genetic data were collected. The genotype was classified into two types: 1) severe mutation genotype: patients who carried at least one of the following three types of mutations: frameshift mutation, splicing mutation, or nonsense mutation; 2) non-severe mutation genotype: patients who only carried missense mutations. Then, the clinical features and genotype of the patients with and without neurological worsening were investigated.

**Results:** Forty-seven neurological WD patients were identified with a median age at onset of 16.17 years (range 7.75–47 years) and 35 (74.5%) males. The mean interval from onset to diagnosis was 0.6 years (range: 0.5 months-6.25 years). Neurological deterioration was observed in 29 patients (61.7%) and the other 18 patients (38.3%) were stable or improved during anti-copper treatment. The neurological worsening was completely irreversible in 6 cases (20.7%) and partially irreversible in 16 cases (55.2%). The common deteriorated symptoms were as follows: rigidity in 20 cases (69%), speech difficulties in 20 cases (69%)), walking difficulties in 13 cases (44.8%), dysphagia in 9 cases (31%), and salivation in 9 cases (31%). The patients with neurological worsening had significantly younger age (*p* = 0.028), shorter delayed diagnosis time (*p* = 0.011), higher rate of dystonia (*p* = 0.003), and severe mutation genotype (*p* = 0.036), compared to those without neurological worsening.

**Conclusion:** We found that younger age of onset, the presence of dystonia, and genotype with severe mutations may be predictive of neurological worsening in the neurological WD patients that received chelator therapy. For those patients, chelator therapy should be given with caution and needs closer observation during follow-up.

## Introduction

Wilson’s disease (WD) is an autosomal recessive inherited disease caused by mutations of *ATP7B* gene, resulting in abnormal copper deposition in multiple organs, predominantly including liver, brain, eyes, and kidneys (Cumings, 1948; Bull et al., 1993; European Association for Study of Liver, 2012). Clinical features vary among the patients and mainly are progressive liver disease and cirrhosis, neuropsychiatric symptoms, Kayser–Fleischer rings in the cornea, and acute liver failure (European Association for Study of Liver, 2012).

Unlike other genetic disorders, WD is one of the few genetic diseases that can be well treated with anti-copper agents, such as chelators of D-penicillamine (DPA), dimercaptopropane sulfonic acid (DMPS), and trientine, which can induce urinary excretion of copper, decrease the copper burden, and thereby relieve the symptoms (Roberts et al., 2008; European Association for Study of Liver, 2012). However, neurological worsening during anti-copper therapy in WD patients has been frequently reported, especially for those with neurological symptoms (Brewer et al., 1987; Walshe and Yealland, 1993; Czlonkowska et al., 1996; Brewer, 1999; Taly et al., 2007; Roberts et al., 2008; European Association for Study of Liver, 2012; Kim et al., 2013; Weiss et al., 2013). Although some studies have tried to investigate whether clinical and laboratory features, and types of anti-copper therapy could affect the occurrence of neurological worsening, no definite association has been established (Prashanth et al., 2005; Weiss et al., 2013; Kalita et al., 2014; Litwin et al., 2015; Ranjan et al., 2015). Some studies have found that severe initial neurological manifestations, the presence of chronic liver disease, leukopenia, thrombocytopenia, and combined use of dopamine receptor antagonists were associated with the neurological worsening in the neurological WD patients (Kalita et al., 2014; Litwin et al., 2015). However, no clinical and laboratory predictors of neurological deterioration were identified in other studies (Prashanth et al., 2005; Weiss et al., 2013). It is worth noting that none of these previous studies have evaluated the role of genotype in the phenomenon of neurological worsening. Besides, the current available studies of genetic analysis in WD patients mostly focus on the relationship between phenotype and genotype, but not the genetic effect on treatment efficacy. None of the available studies have evaluated the genetic predictors of neurological deterioration following treatment.

Considering the high rate of disability owing to neurological worsening, it is important to recognize the potential parameters that may affect the treatment response of chelator in WD patients with cerebral type. Therefore, we conduct the current study to evaluate the clinical and genetic role in the neurological worsening in the neurological WD patients who received initial chelator therapy.

## Materials and Methods

### Patient Population and Data Collection

We screened the medical records of WD patients that were referred to the First Affiliated Hospital of Sun Yat-Sen University between 1992 and 2013. 47 neurological WD patients who had received initial chelating therapy and genetic test were enrolled in the current study. The diagnosis of WD was reviewed using the Leipzig score (Ferenci et al., 2003) and all cases had Leipzig score ≥4. Data of WD characteristics were collected, including age at onset, gender, presence of Kayser–Fleischer rings, neurological symptoms, serum copper, serum ceruloplasmin, 24-h urinary copper at baseline, genotype, chelator therapy, and treatment response. The study was approved by the ethics committee of the First Affiliated Hospital of Sun Yat-Sen University (No [2014] 23).

### Chelator Therapy

All patients have been given chelators as initial therapy. At our center, the protocol is that oral DPA is the first-line treatment for neurological WD patients. If DPA is not tolerated, the patients will receive intravenous DMPS as the second-choice treatment. Both DPA and DMPS were given from low dose and gradually increased to full dose. All patients were followed up closely at least every 3 months after initiation of chelator therapy or when neurological symptoms deteriorated. Compliance was evaluated by telephone and personal interview.

### Treatment Response

The included neurological WD patients were divided into two groups according to treatment response: with or without neurological worsening. All neurological WD patients have been comprehensively evaluated by neurologists and recorded. The severity of neurological symptoms was evaluated by modified Young scale (Zhou et al., 2011). It consists of items evaluating dysarthria, dysphagia, rigidity, ataxia, tremor, choreic movement, gait abnormality, and psychogenia. A higher score indicates a more severe neurological deficit. Neurological worsening was defined as an increase of more than two points in the total score of the modified Young scale. Accordingly, the patients were divided into two groups with and without neurological worsening.

The patients with neurological worsening were analyzed in detail, including the time of neurological worsening from initiation of chelator therapy, specific deteriorated neurological symptoms, and the reversibility of neurological worsening during the follow-up. Then, the comparison between the patients with and without neurological worsening was performed in regarding of age, gender, baseline copper metabolism, genotype, time from symptom onset to treatment initiation, and manifestations and severity of initial neurological deficits.

### Genetic Testing

All enrolled patients have received genetic tests by direct sequencing of all 21 exons within *ATP7B* gene at KingMed Diagnostics (Guangzhou, China). Genomic DNA was extracted from peripheral venous blood samples anticoagulated with ethylenediaminetetraacetic acid. All 21 exons were amplified by polymerase chain reaction (PCR). Direct sequencing of the amplified PCR products was performed using an ABI 3500XL Genetic Analyzer. The sequenced results were aligned to referred ATP7B sequence (NM_000053.3) to figure out the mutations. The ATP7B mutation database (http://www.umd.be/ATP7B) and Human Gene Mutation Database (HGMD, http://www.hgmd.cf.ac.uk/) were referred to identify whether it is a known pathogenic mutation or a novel variant. For all mutations, we applied PolyPhen-2 (http://genetics.bwh.harvard.edu/pph2/) and NetGene2 (http://www.cbs.dtu.dk/services/NetGene2), as appropriate, to predict the putative effect of mutations and identify novel variants as pathogenic mutation or not.

### Statistical Analysis

Results were presented as median with range for continuous variables, or frequency and percentage for categorical variables. All the statistical analyses were conducted using SPSS V.25 (IBM Corporation, New York, United States). Continuous variables were analyzed with unpaired Student t test or the Mann-Whitney U test as appropriate. Categorical variables were analyzed with Fisher’s exact tests. *p* value less than 0.05 was considered signiﬁcant.

## Results

### Demographics

Forty-seven neurological WD patients were identified that received initial chelator therapy and genetic test. As illustrated in Table 1, these patients had a median age at onset of 16.17 years (range 7.75–47 years) with 35 (74.5%) males, and the mean interval from onset to diagnosis was 0.6 years (range: 0.5 months-6.25 years). At the time of diagnosis, the median levels of ceruloplasmin, serum copper, and 24-h urinary copper were 7.28 mg/dl (range 1.7–12 mg/dl), 0.29 mg/L (range 0.06–0.72 mg/L), and 326.6 μg/day (range 87.4–1800 μg/day) respectively. The Kayser–Fleischer ring was observed in 41 (87.2%) patients. The median follow-up period was 2 years (range, 0.5–13 years).

**TABLE 1:** Clinical and laboratory characteristics at the time of diagnosis in 47 neurological WD patients and comparisons between patients with and without neurological worsening.

	All patients	Patients with neurological worsening	Patients without neurological worsening	*p* value
Patient number	47	29	18	
Gender, male, n (%)	35, 74.4%	21, 72.4%	14, 77.8%	0.744
Age at onset, years, median (range)	16.17 (7.75–47)	15.17 (9.5–28.33)	21.71 (7.75–47)	0.028
Time onset to diagnosis, years, median (range)	0.6 (0.04–6.25)	0.5 (0.04–2)	1 (0.08–6.25)	0.011
Kayser-Fleischer ring, n (%)	41, 87.2%	26, 89.7%	15, 83.3%	0.662
Follow-up period, years, median (range)	2 (0.5–13)	3.1 (0.5–10.5)	2 (0.5–13)	0.163
modified Young scale score at baseline (mean ± standard deviation)	9.3 ± 4.0	9.6 ± 3.3	8.9 ± 4.9	0.435
Ceruloplasmin, mg/dL, median (range)	7.28 (1.7–12)	7.25 (1.7–12)	7.28 (2–12)	0.822
Serum copper, mg/L, median (range)	0.29 (0.06–0.72)	0.25 (0.06–0.6)	0.38 (0.2–0.72)	0.054
Urinary copper, μg/day, median (range)	326.6 (87.4–1800)	300 (87.4–1800)	364.7 (109–1358.7)	0.257

### Treatment Response Analysis

All of the 47 neurological WD patients received oral DPA as initial chelator therapy and 46 of them were well tolerated with DPA. One patient discontinued DPA due to fever and rash, and subsequently received intravenous DMPS. For the treatment response, neurological worsening was observed in 29 patients (61.7%, Group 1) and the other 18 patients (38.3%, Group 2) were stable or improved during anti-copper treatment. The patient that had received DMPS was in the group without neurological worsening.

Seven patients in Group 1 showed improvement before deterioration within 0.5–6 months after initiation of the chelator therapy. The main improved symptoms were speech, gait, and tremor. The other 22 patients in Group 1 showed no improvement before neurological deterioration. The median duration from initiation of anti-copper treatment to neurological deterioration was 2 months (range: 0.5–14 months). In Group 2, 3 patients stayed stable on chelator and 15 patients had improvements within 0.25–12 months after initiation of chelator therapy. The main improved symptoms were tremor, involuntary movement, and gait.

For the patients with neurological worsening in Group 1, the common deteriorated symptoms were as follows: rigidity in 20 cases (69%), speech difficulties in 20 cases (69%)), walking difficulties in 13 cases (44.8%), dysphagia in 9 cases (31%), and salivation in 9 cases (31%). Other uncommon, deteriorated symptoms included tremor in 6 cases (20.7%) and psychogenia in 2 cases (6.9%). Eleven patients (37.9%) developed new neurological symptoms after initiation of chelator therapy, which were mainly slurred speech, dysphagia, salivation, and walking difficulties. During the follow-up, the neurological worsening was completely irreversible in 6 cases (20.7%), partially irreversible in 16 cases (55.2%), and completely reversible in 7 cases (24.1%). 12 patients (41.4%) that experienced neurological deterioration remained severely disabled and could not live independently.

### Clinical and Genetic Comparisons of WD Patients With and Without Neurological Worsening

As illustrated in Table 1, the patients with neurological worsening in Group 1 had a significantly younger age at onset (*p* = 0.028) and shorter delayed diagnosis time (*p* = 0.016), compared to those without neurological worsening in Group 2. Some data were available for only a subset of patients owing to the retrospective nature of the study. However, no statistically significant differences were found between the patients in Group 1 and Group 2 in regarding of gender, ceruloplasmin, serum copper, 24-h urinary copper, and severity of neurological deficit at baseline (*p* > 0.05). Eight patients in Group 1 and 3 patients in Group 2 had poor compliance, and there was no significant difference (*p* > 0.05).

In respect of neurological symptoms, dystonia was significantly more frequently seen in patients with neurological worsening than those without (96.6 vs. 61.1%, *p* = 0.003). For the symptoms of tremor and gait abnormality, no statistically significant differences were found between the patients with and without neurological worsening (*p* > 0.05). Quantitative analysis of other neurological symptoms was not possible due to their small number.

There was no significant difference in regarding of the ratio of homozygotes mutation between the patients with and without neurological worsening (*p* > 0.05). For the ratio of the most common mutations of p.Pro992Leu and p.Arg778Leu, no difference was found between the two groups (*p* > 0.05). Frameshift mutation, splicing mutation, and nonsense mutation were considered more seriously affected the function of ATP7B protein than that of missense mutation. Thereby, we classified the patients’ genotype into two groups: 1) severe mutation genotype: patients who carried at least one of the following three types of mutations: frameshift mutation, splicing mutation, and or nonsense mutation; 2) non-severe mutation genotype: patients who only carried missense mutations. The patients with neurological worsening harbored significantly more severe mutation genotype than those without neurological worsening (55.2 vs. 22.2%, *p* = 0.036, Table 2).

**TABLE 2:** Comparison of genotype in patients with and without neurological worsening.

Genotype	Patients with neurological worsening	Patients without neurological worsening	*p* value
Severe mutation genotype	16	4	0.036
Non-severe mutation genotype	13	14	

### Mutation Analysis

The enrolled 47 neurological WD patients were derived from 47 unrelated families. 44 patients had pathogenic mutations in both alleles (9 homozygotes and 35 compound heterozygotes), 2 patients had mutations in one allele, and 1 patient had three variants which were homozygotes of c.3443T > C (p.Ile1148Thr) and heterozygote of c.3426G > C (p.Gln1142His). The overall mutation detection rate was 97.9%.

**FIGURE 1:**
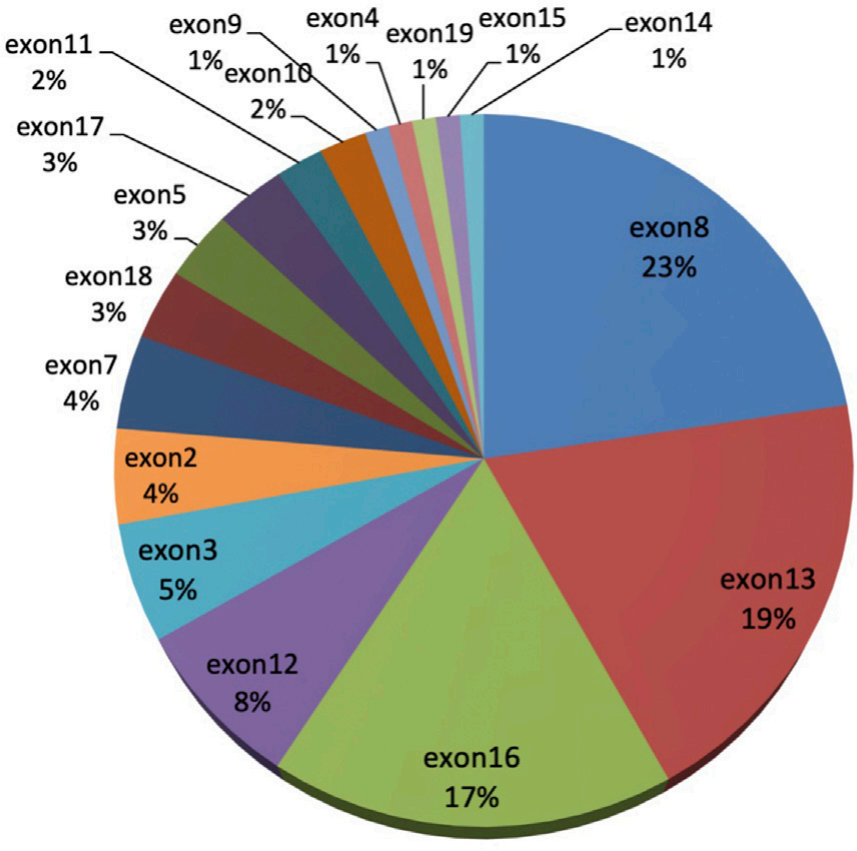
The distribution of ATP7B gene mutations in 47 neurological WD patients.

Among the 47 WD patients, a total of 34 different mutations (21 missense, 3 nonsense, 3 insertion, 1 deletion, and 6 splicing) were detected (Table 3). Four novel pathogenic mutations were identified: c.3917A > T (p.Asp1306Val), c.166C > T (p.Gln56*), c.1682delG (p.Gly561Alafs*8), and c.3087dupT (p.Gly1030Trpfs*39).The most common mutation was p.Pro992Leu (19.2%), followed by p.Arg778Leu (12.8%), p.Ile1148Thr (10.64%), p.Met769Hisfs*26 (7.5%), and p.Gly943Asp (6.4%). These five mutations accounted for 56.4% of all mutant alleles. The exons harboring the highest percentage of mutations were exon 8 (22.6%), exon 13 (19.4%), exon 16 (17.2%), exon 12 (7.5%), and exon 3 (5.4%) (Figure 1). The total mutation detection rate within these five exons was 72%.

**TABLE 3:** Spectrum and frequency of ATP7B gene mutations in 47 neurological WD patients.

Mutation	Exon	Amino acid change	Domain	Homo-zygote	Hetero-zygote	Allelic frequency (%)
Missense						
c.1847G > A	5	p.Arg616Gln	Cu6	0	1	1.06
c.2077T > C	7	p.Ser693Pro	TM2	1	0	2.13
c.2078C > G	7	p.Ser693Cys	TM2	0	1	1.06
c.2293G > A	8	p.Asp765Asn	TM4	0	1	1.06
c.2333G > T	8	p.Arg778Leu	TM4	2	8	12.77
c.2480G > A	10	p.Arg827Gln	bet TM4/Td	0	1	1.06
c.2549C > T	10	p.Thr850Ile	Td	0	1	1.06
c.2621C > T	11	p.Ala874Val	bet Td/TM5	0	1	1.06
c.2662A > C	11	p.Thr888Pro	bet Td/TM5	0	1	1.06
c.2755C > G	12	p.Arg919Gly	bet TM6/Ph	0	1	1.06
c.2828G > A	12	p.Gly943Asp	TM5	0	6	6.38
c.2975C > T	13	p.Pro992Leu	bet TM6/Ph	4	10	19.15
c.3426G > C	16	p.Gln1142His	ATP-loop	0	1	1.06
c.3443T > C	16	p.Ile1148Thr	ATP-loop	1	8	10.64
c.3445G > A	16	p.Gly1149Arg	ATP-loop	0	1	1.06
c.3517G > A	16	p.Glu1173Lys	ATP-loop	0	2	2.13
c.3532A > G	16	p.Thr1178Ala	ATP-loop	0	1	1.06
c.3646G > A	17	p.Val1216Met	ATP bind	1	2	4.26
c.3818C > A	18	p.Pro1273Gln	ATP-hinge	0	2	2.13
c.3818C > T	18	p.Pro1273Leu	ATP-hinge	0	1	1.06
c.3917A > T	19	p.Asp1306Val	bet ATP hinge/TM7	0	1	1.06
Nonsense						
c.166C > T	2	p.Gln56*	Cu1	0	1	1.06
c.1470C > A	3	p.Cys490*	Cu5	0	1	1.06
c.1531C > T	3	p.Gln511*	Cu5	0	2	2.13
Insertion						
c.525dupA	2	p.Val176Serfs*28	Cu2	0	3	3.19
c.2304dupC	8	p.Met769Hisfs*26	TM4	1	5	7.45
c.3087dupT	14	p.Gly1030Trpfs*39	Ph	0	1	1.06
Deletion						
c.1682delG	4	p.Gly561Alafs*8	Cu6	0	1	1.06
Splicing						
c.1543+1G > T	3	NA	Cu5	0	2	2.13
c.1708-1G > C	5	NA	Cu6	0	2	2.13
c.2122–8T > G	8	NA	TM2	0	1	1.06
c.2356–2A > G	9	NA	bet TM4/Td	0	1	1.06
c.3244–2A > G	15	NA	ATP-loop	0	1	1.06
c.3556+1G > A	16	NA	ATP-loop	0	1	1.06

ATP-loop, ATP-loop domain; ATP-hinge, ATP-hinge domain; bet, between; Cu, copper-binding domain; Ph, Phosphorylation domain; TM, transmembrane domain; Td, transduction domain.

## Discussion

The phenomenon of neurological worsening during anti-copper therapy has been frequently reported in WD, especially for neurological type. However, it is still not clear which subtypes of the patients are prone to experience neurological worsening. There is no previous study that investigated the role of genotype in this phenomenon.

In our study, we found that neurological worsening was observed in 61.7% of patients. Meanwhile, the patients with neurological worsening had a younger age at onset, shorter delayed diagnosis, and higher frequency of dystonia and severe mutation genotype, compared to the patients without neurological worsening. It is indicated that these factors may associated with the occurrence of neurological worsening.

We observed that 61.7% of patients experienced neurological worsening after receiving chelator therapy. This is relatively higher than the previously reported percentage of neurological worsening which ranges from 3 to 52% (Brewer et al., 1987; Czlonkowska et al., 1996; Prashanth et al., 2005; Weiss et al., 2013). One important reason is that all of our enrolled WD patients were neurological type, while the previous studies enrolled different subtypes of WD patients, including asymptomatic, and hepatic types. It has been reported that neurological worsening mostly occurred in neurological WD patients (Weiss et al., 2013; Ranjan et al., 2015). Thereby, the percentage of neurological worsening was relatively higher in our study. Besides, the retrospective nature of our study is another unignorable factor that may contribute to the high rate of neurological worsening, since the patients with good treatment response were prone to be lost to follow-up.

It is unexpected that the patients with neurological deterioration had a younger age and shorter time of delayed diagnosis. This might be related to the pathogenesis of WD. WD is a genetic disorder characterized by the accumulation of excessive amounts of copper, which is a dynamic progressing process of gradual copper accumulation (European Association for Study of Liver, 2012). The WD patients with cerebral type were generally in the period of extensive copper accumulation in multiple organs including the brain. Currently, the mechanism of the neurological worsening during anti-copper therapy in neurological WD patients is still poorly understood. The rapid mobilization of copper during anti-copper therapy and transiently increased amount of toxic free copper in serum and brain were suggested to increase copper-related damage in the brain and lead to neurological worsening (Brewer et al., 1987; Brewer, 1999; Stuerenburg, 2000; European Association for Study of Liver, 2012). Besides, a previous animal study of WD found that the pathological characteristics of the brains of toxic milk mice were different at different stages, that copper deposition at an early stage, copper and iron deposition in the middle stage, and abnormal oxidative stress in the late stage (Zhou et al., 2019). The patients with younger age and shorter time of delayed diagnosis meant that they had a shorter period of copper accumulation and neurological toxicity of copper. At this period, we speculate that the neurons may be still in the active injury stage and relatively more sensitive to copper toxicity. Thereby, the patients with younger age and shorter time of delayed diagnosis may be more sensitive to copper redistribution and the resulting copper-related oxidative stress caused by chelator therapy and probably more likely to experience neurological worsening. The patients who had an elder age and longer time of delayed diagnosis, experienced a longer period of copper deposition and copper toxicity before decoppering therapy, which may enable the brain tissue more tolerant to the toxicity of copper redistribution. However, this is our speculation, and it needs to be verified in further study. Prashanth et al. and Walshe et al. have investigated the prognosis of neurological WD patients on chelator therapy, but they observed that delayed diagnosis did not influence the outcome (Walshe and Yealland, 1992; Prashanth et al., 2005).

On the other hand, an earlier MRI study in WD patients found that the patients with a longer duration of untreated disease and overall duration of disease had significantly less severe changes in signal intensity on MRI (King et al., 1996). Younger age and shorter time of delayed diagnosis mean a shorter course of the disease. Therefore, these patients were supposed to have more severe changes of the lesion on MRI and probably more susceptible to neurological worsening.

The current genetic studies in WD patients mostly focus on the relationship between phenotype and genotype, but not the genetic effect on treatment efficacy. Till now, over 900 pathogenic mutations in the *ATP7B* gene have been identified in WD patients (Couchonnal et al., 2021). Most of the patients are compound heterozygotes with different mutations on each of the *ATP7B* alleles. In our study, only 10 patients were found to be homozygotes, and 34 different mutations were identified. We also identified four novel mutations in *ATP7B* gene. For the ratio of homozygotes and most common mutations (p.Pro992Leu and p.Arg778Leu), no difference was found between the patients with and without neurological worsening. The very high allelic heterogeneity limited the analysis of the specific mutation. However, in regarding of mutation types according to the dysfunctional severity of mutant ATP7B protein, we found that the neurological WD patients with severe mutation genotype may be more likely to experience neurological worsening during anti-copper treatment. In our study, we define nonsense mutation, frameshift mutation, and splicing mutation as severe mutation, as these types of mutation are expected to profoundly affect the function of ATP7B protein due to the absence of a full-length gene product, which was typically associated with complete loss of normal structure and function of ATP7B protein (Panagiotakaki et al., 2004; Gromadzka et al., 2005). As a result, severe mutations would lead to more severe disturbances of copper metabolism, which may present as much lower ceruloplasmin, free copper, and higher 24-h urinary copper (Panagiotakaki et al., 2004; Gromadzka et al., 2005; Couchonnal et al., 2021). Therefore, the patients with these severe mutations were probably not capable of well responding to copper redistribution following chelator therapy and this may cause more severe copper toxicity injury. Accordingly, these underlying conditions would probably place those patients at high risk of neurological worsening. To the best of our knowledge, this is the first study that reported the association between the type of mutations and neurological worsening in neurological WD patients. However, in Weiss et al.‘s cohort, they did not find that the phenomenon of neurological worsening was associated with genotype (Weiss et al., 2013). Unfortunately, the data was not shown. It was not known how the analysis of genotype was performed. Besides, epigenetic and other genetic factors may also play a role in the treatment response (Medici and Weiss, 2017). More studies were needed to evaluate whether the genotype or variations in modifier loci could affect the treatment response in different subtypes of WD patients.

In the neurological WD patients, the common symptoms were dystonia, rigidity, tremor, slurred speech, and gait disturbance in our study and previous reports (Walshe and Yealland, 1992; Machado et al., 2006; Taly et al., 2007). We found that the patients with neurological worsening exhibited a significantly higher ratio of dystonia than those without neurological worsening. Meanwhile, it is interesting that dystonia and dysarthria have been also reported as the most refractory to anti-copper treatment (Denny-Brown, 1964; Walshe and Yealland, 1993; Czlonkowska et al., 1996; Pellecchia et al., 2003; Arnon et al., 2007; Burke et al., 2011). A study has observed that dystonia and abnormal facial expression were the features that improved least over the duration of follow-up, while tremor was the most favorable symptom during anti-copper treatment (Burke et al., 2011). Therefore, dystonia is both a prognostic sign of neurological worsening and amongst the key features most resistant to anti-copper treatment.

The severity of initial neurological manifestations may also play a role in the occurrence of neurological worsening. Litwin et al. has reported that WD patients who have advanced neurological symptoms were more likely to experience neurological worsening (Litwin et al., 2015). They also found that patients with early deterioration more frequently present with thalamic and brain stem lesions, which were considered to be severe brain lesions (Prayer et al., 1990). Ranjan et al.‘s study on the MRI changes in WD patients with neurological worsening found that the deterioration was associated with the appearance of new lesions on MRI especially involving white matter (Ranjan et al., 2015). Since MRI is a sensitive method for the detection and assessment of brain abnormalities in WD, several MRI scales have been investigated recently to facilitate the evaluation of disease severity and treatment outcome (Poujois et al., 2017; Dusek et al., 2020). Dusek et al. developed a novel scale that consisted of acute toxicity and chronic damage subscores, and they found that chronic damage and the total score were positively associated with the disease severity (Dusek et al., 2020). It is indicated that validated neuroimaging scales can be used for standardized monitoring of chelator therapy in clinical practice and future therapeutic studies.

In addition to clinical and neuroimaging scales, serum biomarkers have been explored in WD patients for the potential use in evaluating disease severity and guiding anti-copper treatment, especially for neurological WD patients, such as neuroﬁlament light chain (NFL), glial ﬁbrillary acidic protein, ubiquitin carboxyl-terminal hydrolase L1, and tau protein. Recent studies found that serum NFL concentration was significantly higher in neurological WD patients, compared to hepatic and control groups. Moreover, serum NFL concentrations were positively associated with active neurological disease, clinical neurological severity scores, and brain MRI severity scores (Shribman et al., 2021a; Ziemssen et al., 2022). These findings highlight the potential value of NFL as a novel approach to evaluate brain injury, monitor treatment efficacy, and predict neurological worsening in WD patients.

In the group of neurological worsening, most of the cases experienced neurological worsening within 0.5–3 months from initiation of WD anti-copper treatment. This is consistent with the previous studies that the phenomenon of neurological worsening mostly occurred in the early stage of anti-copper treatment, which could be supported by the above-mentioned copper redistribution theory (Brewer et al., 1987; Brewer, 1999; Stuerenburg, 2000; Weiss and Stremmel, 2012; Litwin et al., 2015). Three patients in our study exhibited neurological worsening 6 months after initiation of anti-copper treatment. It is uncommon for late neurological deterioration, and it is reported that this is associated mainly with poor adherence (Czlonkowska et al., 2005; Masełbas et al., 2010). One of the above three patients with late neurological deterioration in our study had poor compliance. It has been reported that 25–32% of WD patients have low adherence to the treatment, which is very challenging for the long-term management of WD patients (Maselbas et al., 2019a; Maselbas et al., 2019b; Jacquelet et al., 2021). There is no doubt that non-compliance has an important negative impact not only on clinical outcomes of WD patients, but also on education level and work ability, regardless of the disease form (Maselbas et al., 2019b). Maselbas et al. has reported that clinical worsening was noted in 52.2% of treatment non-persistent patients with WD (Maselbas et al., 2019a). Several factors may help improve treatment adherence of WD patients including family support, higher education, multidisciplinary management on a case-by-case basis, available medicine from community pharmacies, and once-daily dosage (Maselbas et al., 2019a; Jacquelet et al., 2021).

During the follow-up, we found that the neurological deterioration was completely irreversible in 20.7% of patients and lead to a high rate of severe disability (41.4%), which is in line with the previous studies (Litwin et al., 2015). Therefore, it is important to recognize the potential parameters that may influence the treatment response of chelator in WD patients, especially for those with neurological symptoms. Our study indicated that the younger age of onset, the presence of dystonia, and genotype with severe mutations may play a role in the occurrence of neurological deterioration in neurological WD patients who received chelator therapy. For those patients, it may be helpful to pay more attention to the choice of therapy and close observation of efficacy. The principle of “start low and go slow” for the use of chelators in WD patients should be kept in mind. However, stronger and faster anti-copper therapy, which could remove brain copper more quickly, and may could have a better clinical outcome in these patients. There are several potential new therapeutic approaches in recent years, such as novel chelating agents, gene therapy, and targeted molecular therapy (Shribman et al., 2021b). Bis-choline tetrathiomolybdate (TTM), a novel promising copper-protein-binding agent, is capable of rapidly controlling free copper and detoxifying circulating reactive copper in tripartite complexes due to its unique mechanism of action. A phase II study of Bis-choline TTM showed significant early improvements in neurological symptoms (Weiss et al., 2017). More attractively, no cases of early neurological worsening were observed in both previously treated patients and treatment naive patients in this study. It is postulated that the binding of Bis-choline TTM to copper in an inert protein complex that cannot redistribute to the brain, contributed to the apparent absence of early chelator-induced neurologic deterioration (Rupp et al., 2017; Weiss et al., 2017). Another recent study of susceptibility-weighted imaging in patients with WD found that the combined use of DMPS and dimercaptosuccinic acid can remove metal from brain tissue faster than DPA and lead to less neurological worsening, compared to DPA (Zhou et al., 2020).

## Limitation

This study had several limitations. First, there may be some bias due to the retrospective nature of the study. Selection bias may present since the patients with good treatment response were prone to be lost to follow-up. This may partially explain the relatively small number of patients who did not have neurological deterioration in our study. Besides, laboratory parameters were available for only a subset of patients. Second, compliance was only evaluated by telephone and personal interview without objective parameters, such as 24-h urinary copper during the chelator therapy. Third, we did not evaluate the effect of the concomitant use of drugs that relieve neurological symptoms, such as neuroleptics and anti-emetics. It has been reported that these anti-dopaminergic drugs may be related to neurological worsening after initiation of chelator therapy (Litwin et al., 2015). Forth, the potential interaction of the possible prognostic factors of neurological worsening that we identified in our study was not further analyzed owing to the limitation of the small sample size.

## Conclusion

In conclusion, we identified four novel mutations in *ATP7B* gene and found that younger age of onset, the presence of dystonia, and genotype with severe mutations may be predictive of neurological worsening in the neurological WD patients that received chelator therapy. For those patients, chelator therapy should be given with caution and needs closer observation during follow-up. More studies with a large cohort are needed to further investigate the prognostic factors of neurological worsening and optimize anti-copper therapy in neurological WD patients.

## Data Availability

The original contributions presented in the study are included in the article/Supplementary Material, further inquiries can be directed to the corresponding authors.
